# Is the prognosis of non‐hypertensive, COVID‐19 patients treated with renin–angiotensin–aldosterone system inhibitors more uncertain?

**DOI:** 10.14814/phy2.15512

**Published:** 2022-11-17

**Authors:** Juan José García Martínez, Hannah Wozniak, Pauline Salamin, Raphaël Giraud, Christophe Le Terrier, Karim Bendjelid

**Affiliations:** ^1^ Intensive Care Unit Hôpital du Valais Sion Switzerland; ^2^ Intensive Care Division Geneva University Hospitals Geneva Switzerland; ^3^ Geneva Hemodynamic Research Group Geneva Switzerland; ^4^ Faculty of Medicine Geneva University Hospitals Geneva Switzerland

**Keywords:** ACE2, COVID‐19, hypertension, ICU, renin–angiotensin–aldosterone

## Abstract

Previous studies suggested that ongoing treatment with renin–angiotensin–aldosterone system (RAAS) inhibitor drugs may alter the course of SARS‐CoV‐2 infection and promote the development of more severe forms of the disease. The authors conducted a comparative, observational study to retrospectively analyze data collected from 394 patients admitted to ICU due to SARS‐CoV‐2 pneumonia. The primary aim of the study was to establish an association between the use of RAAS inhibitor drugs and mortality in the ICU. The secondary aims of the study were to establish an association between the use of RAAS inhibitor drugs and clinical severity at ICU admission, the need for tracheal intubation, total days of mechanical ventilation, and the ICU length of stay. The authors found no statistically significant difference in ICU mortality between patients on RAAS inhibitor drugs at admission and those who were not (31.3% versus 26.2% mortality, *p*‐value 0.3). However, the group of patients taking RAAS inhibitor drugs appeared to be more critical at ICU admission, and this difference became statistically significant in the subgroup of non‐hypertensive patients. ICU mortality in the subgroup of non‐hypertensive patients treated with RAAS inhibitor drugs also tended to be higher. Overexpression of the angiotensin‐converting enzyme 2 (ACE2) in human cells, induced by RAAS inhibitor drugs, promotes viral entry‐replication of SARS‐CoV‐2 and alters the basal balance of the RAAS, which may explain the findings observed in the present study. These phenomena may be amplified in non‐hypertensive patients treated with RAAS inhibitor therapy.

## Graphical abstract

Overexpression of the angiotensin‐converting enzyme 2 (ACE2) in human cells, induced by RAAS inhibitor drugs, promotes viral entry‐replication of SARS‐CoV‐2 and alters the basal balance of the RAAS, which may explain the more severe course of COVID‐19 suggested by the results of this study. These phenomena may be amplified in non‐hypertensive patients treated with RAAS inhibitor therapy.

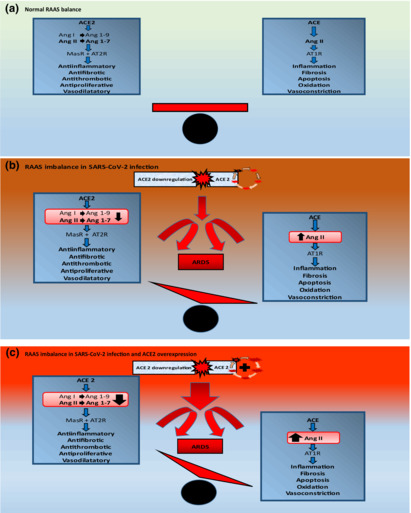

## 1 INTRODUCTION

The SARS‐CoV‐2 pandemic has caused several million deaths worldwide. Major efforts are underway to discover effective treatments for the virus and the disease it produces (i.e., COVID‐19).

In a scenario where SARS‐CoV‐2 becomes endemic, it is likely that some patients would still develop severe forms of the disease, such as bilateral interstitial pneumonia and ARDS, and it is important to identify manageable factors that could influence this unfavorable clinical course. This knowledge could also be applied to other coronaviruses now and in the future.

Basic research has already demonstrated that the angiotensin‐converting enzyme 2 (ACE2) serves as a specific target for SARS‐CoV‐2 and that the lung appears to be the most vulnerable target organ (Zhang et al., 2020). Moreover, SARS‐CoV‐2 and others SARS‐CoV likely cause downregulation of surface ACE2 expression (Gross et al., 2020) and lead to decreased availability of anti‐inflammatory, anti‐fibrotic, and vasodilatory angiotensin 1–9 (Ang 1–9) and angiotensin 1–7 (Ang 1–7) fragments and to unopposed angiotensin II (Ang II) accumulation, which may accelerate the progression of lung injury and COVID‐19 disease (Cousin et al., 2021).

Pending the development of effective treatments for COVID‐19, clinical data are needed to provide a basis for further research. Considering that ACE2 is overexpressed in patients on RAAS inhibitor therapy, the hypothesis that RAAS inhibitor drugs may alter the risk of developing more severe forms of COVID‐19 by facilitating viral entry and replication in human cells and altering the basal balance of RAAS is a matter of debate (Baral et al., 2020; Kaur et al., 2020; Zheng et al., 2020).

In the present bicentric study, we aimed to investigate whether a relationship exists between the use of RAAS inhibitor drugs and the severity and lethality of COVID‐19.

## 2 MATERIALS AND METHODS

We conducted a comparative, observational study to retrospectively analyze data collected from the records of patients hospitalized in adult ICUs of the Geneva University Hospitals (Geneva, Switzerland) and the Sion Hospital (Valais Hospital, Sion, Switzerland) between 01.03.2020 and 31.05.2021 due to SARS‐CoV‐2 pneumonia. We excluded patients who tested positive for SARS‐CoV‐2 but were admitted to the ICU for another medical reason unrelated to SARS‐CoV‐2 pneumonia as well as patients transferred to other units and/or hospitals during their stay and having incomplete data. Patients on RAAS inhibitor therapy at hospital admission were compared with patients not taking RAAS inhibitor drugs. The primary aim of the study was to establish an association between the use of RAAS inhibitor drugs and ICU mortality in patients admitted due to SARS‐CoV‐2 pneumonia. The secondary aims of the study were to establish an association between the use of RAAS inhibitor drugs and clinical severity at ICU admission (determined by the Simplified Acute Physiology Score – *SAPS II)*, the need for tracheal intubation, the total days of mechanical ventilation, and the ICU length of stay (LOS).

For data analysis, the Mann–Whitney U test was used to compare continuous variables between both groups. Chi‐squared or Fisher's exact test was used for categorical variables as appropriate. To investigate the association between RAAS inhibitor drugs and ICU mortality, we performed a multivariable logistic regression adjusting the estimates for SAPS II and the presence of hypertension, heart disease, or diabetes mellitus in the clinical history of the patient. The results of the logistic regression are expressed as odds ratios (OR) and 95% confidence intervals (CI 95%). Two‐tailed *p*‐values ≤0.05 were considered statistically significant. Analyses were performed using Stata® IC 16.0 (StataCorp, TX, USA).

## 3 RESULTS

Of the 394 patients admitted to the adult ICUs of the Geneva University Hospitals and the Sion Hospital during the study period due to SARS‐CoV‐2 pneumonia, 150 patients (38.1%) were on RAAS inhibitor therapy at the time of hospital admission. Patients on RAAS inhibitor therapy were older (median age 71 yrs. versus 65 years, *p* < 0.01), had a higher prevalence of diabetes mellitus (36.7% versus 25.0%, *p* < 0.02), hypertension (90.7% versus 22.1%, *p* < 0.01), or cardiomyopathy (32.7% versus 10.7%, *p* < 0.01) than patients not taking a RAAS inhibitor.

ICU mortality was 26.2% in the non‐RAAS inhibitor group compared with 31.3% in the RAAS inhibitor group. In univariate analysis, no statistically significant differences were found in ICU mortality, the need for tracheal intubation, total days of mechanical ventilation, or LOS in the ICU between patients on RAAS inhibitors at admission and those who were not (Table 1). After adjustment for patient severity by SAPS II score and the presence of hypertension, heart disease, or diabetes mellitus in the clinical histories of patients, the use of RAAS inhibitor drugs was not associated with mortality (Table 2).

**TABLE 1:** Characteristics and outcomes of all patients

*N* total = 394	No RAAS inhibitor therapy *n* = 244	RAAS inhibitor therapy *n* = 150	*p*‐value
Admission (*n*, %)			
Geneva University Hospitals	168 (68%)	88 (58.7%)	
Sex (*n*, %)			
Women	71 (29.1%)	37 (24.7%)	0.3
Age (median, IQR)	65.2 (57–73)	71.1 (64.4–77)	<0.01
Diabetes (*n*, %)	65 (25.8%)	55 (36.7%)	0.02
Hypertension (*n*, %)	54 (22.1%)	136 (90.7%)	<0.01
Cardiomyopathy (*n*, %)	26 (10.7%)	49 (32.7%)	<0.01
SAPS II (median, IQR)	43.5 (30–59)	44 (35–64)	0.08
APACHE II (median, IQR)	22 (13–28)	23.5 (15–30)	0.2
Tracheal intubation (*n*, %)	178 (72.9%)	108 (72%)	0.8
Time from hospital admission to tracheal intubation in days (median, IQR)	3 (1–6)	3 (1–6)	0.8
Mechanical ventilation duration in days (median, IQR)	11 (5–18)	10.7 (4–17)	0.6
Hospital length of stay in days (median, IQR)	24 (14–34.5)	26 (15–37)	0.3
Length of stay in the ICU in days (median, IQR)	12 (5–20.5)	13 (7–22)	0.2
Mortality in the ICU (*n*, %)	64 (26.2%)	47 (31.3%)	0.3

Abbreviations: *APACHE II*, Acute Physiology And Chronic Health Evaluation II; RAAS, renin–angiotensin–aldosterone system; SAPS II, Simplified Acute Physiology Score *II*.

**TABLE 2:** RAAS inhibitor drug use and ICU mortality

All ICU patients		
*N* = 394	Mortality in the ICU, OR (CI 95%)	*p*‐value
RAAS inhibitor therapy	1.2 (0.6–2.2)	0.5
SAPS II	1.05 (1.03–1.06)	<0.01
Hypertension	0.8 (0.4–1.5)	0.4
Diabetes	1.4 (0.8–2.3)	0.3
Cardiomyopathy	1.4 (0.8–2.6)	0.2

Abbreviations: RAAS, renin–angiotensin–aldosterone system; SAPS II, Simplified Acute Physiology Score *II*.

**FIGURE 1:**
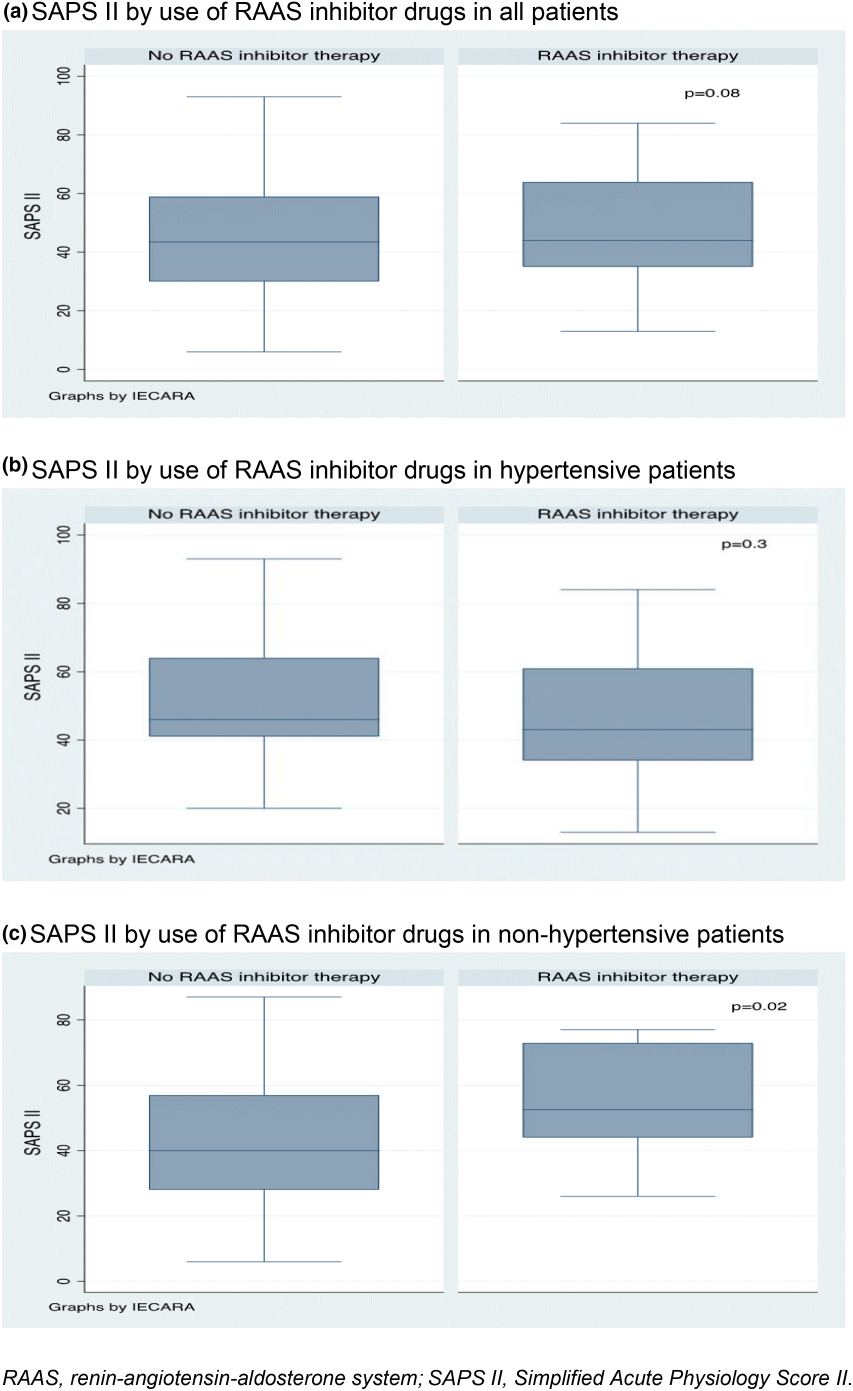
Severity at ICU admission and the use of RAAS inhibitor drugs. (a) SAPS II by use of RAAS inhibitor drugs in all patients. (b) SAPS II by use of RAAS inhibitor drugs in hypertensive patients. (c) SAPS II by use of RAAS inhibitor drugs in non‐hypertensive patients. RAAS, renin–angiotensin–aldosterone system; SAPS II, Simplified Acute Physiology Score II.

However, the group of patients taking RAAS inhibitor drugs appears to be more critical at ICU admission (median SAPS II of 44 pts versus 43.5 pts in the non‐RAAS inhibitor group, *p* 0.08). This difference becomes statistically significant in the subgroup of non‐hypertensive patients (median SAPS II of 52.5 pts for patients on RAAS inhibitor therapy versus 40 pts in the non‐RAAS inhibitor group, *p* 0.02) (Table 3; Figure 1).

**TABLE 3:** Characteristics and outcomes of non‐hypertensive patients

*N* total = 204	No RAAS inhibitor therapy *n* = 190	RAAS inhibitor therapy *n* = 14	*p‐*value
Admission (*n*, %)			
Geneva University Hospitals	130 (68.4%)	8 (57.1%)	
Sex (*n*, %)			
Women	56 (29.5%)	2 (14.3%)	0.4
Age (median, IQR)	62 (55–72.7)	75.5 (65.1–77.6)	0.01
Diabetes (*n*, %)	36 (19%)	3 (21.4%)	0.8
Cardiomyopathy (*n*, %)	16 (8.4%)	9 (64.3%)	<0.01
SAPS II (median, IQR)	40 (28–57)	52.5 (44–73)	0.02
APACHE II (median, IQR)	21 (12–28)	23 (14–32)	0.4
Tracheal intubation (*n*, %)	138 (72.6%)	12 (85.7%)	0.4
Time from hospital admission to tracheal intubation in days (median, IQR)	3 (1–6)	3 (0–5)	0.6
Mechanical ventilation duration in days (median, IQR)	11 (5–19)	10.7 (3.7–13)	0.3
Hospital length of stay in days (median, IQR)	24 (14.5–35)	19 (12.5–35)	0.5
Length of stay in the ICU in days (median, IQR)	12 (6–22)	12.5 (5–19)	0.7
Mortality in the ICU (*n*, %)	47 (24.7%)	7 (50%)	0.06

Abbreviations: *APACHE II*, Acute Physiology And Chronic Health Evaluation II; AAS, renin–angiotensin–aldosterone system; SAPS II, Simplified Acute Physiology Score *II*.

Moreover, ICU mortality in the subgroup of non‐hypertensive patients treated with RAAS inhibitors tended to be higher (50% mortality versus 24.7% in patients without RAAS inhibitor therapy, *p* 0.06).

## 4 DISCUSSION

The present study demonstrates no statistically significant difference in ICU mortality between patients on RAAS inhibitor drugs at admission and those who were not. This result was confirmed after adjustment for potential confounding factors. The study also demonstrates no statistical significant difference in the need for tracheal intubation, total days of mechanical ventilation, or the ICU LOS. However, the group of patients taking RAAS inhibitors appears to be more critical at ICU admission, and this difference becomes statistically significant in the subgroup of non‐hypertensive patients. Moreover, mortality in this latter subgroup of patients treated with RAAS inhibitor drugs also tends to be higher.

ACE2 is a membrane‐bound aminopeptidase that acts as a negative regulator of RAAS and localizes to the cell surface in the renal, cardiac, pulmonary, and gastrointestinal systems. ACE2 cleaves angiotensin I (Ang I) and angiotensin II (Ang II) into the vasodilator fragments angiotensin 1–9 and angiotensin 1–7, respectively, while decreasing the concentration of Ang II to further facilitate vasodilatory and anti‐hypertensive effects (Patel et al., 2016). Angiotensin 1–7 has anti‐inflammatory, antifibrotic, and vasodilatory effects through mitochondrial assembly receptor (MasR), alamandine‐MrgD‐TGR7 (Gs) pathway and angiotensin type 2 receptor (AT2R) activation whereas Ang II has proinflammatory and profibrotic activity after angiotensin type 1 receptor (AT1R) binding (Gheblawi et al., 2020; Kaur et al., 2020).

A protective role for ACE2 in ARDS has been proposed in the past (Imai et al., 2008). Epithelial injury is the cornerstone of ARDS and may result in impaired ACE2 activity and dominant ACE action leading to increased pulmonary permeability, local production of inflammatory cytokines, alveolar epithelial cell apoptosis, and lung fibroproliferation. A crucial role of ACE2 has been suggested in the development of lung injury in the context of the SARS‐CoV outbreak of 2003 (Kuba et al., 2005), and the use of recombinant ACE2 (rACE2) protein has been reported to have a protective effect on ARDS in animal models (Zou et al., 2014). The role of ACE2 as a receptor for SARS‐CoV was described years ago and confirmed for SARS‐CoV‐2 by several authors (Li et al., 2003; Yan et al., 2020). Binding of the viral spike protein to the extracellular domains of transmembrane ACE2 proteins in target cells allows the virus to enter and infect cells. This binding is thought to result in downregulation of ACE2 activity and an imbalance in previous ACE and ACE2 signaling (Cousin et al., 2021) with a loss of the protective effect of ACE2.

**FIGURE 2:**
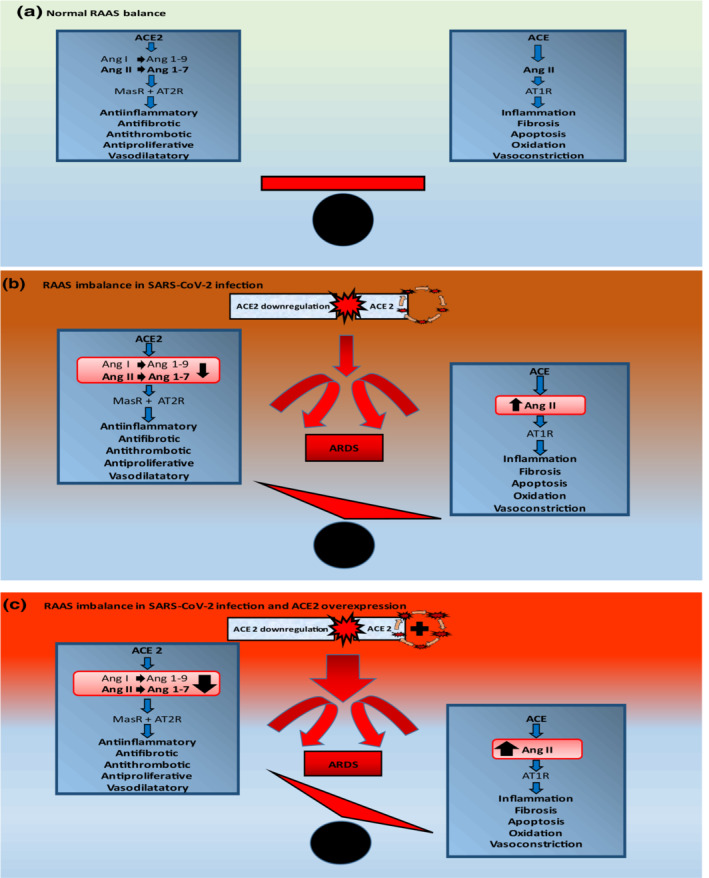
RAAS balance and SARS‐CoV‐2 infection. Renin–angiotensin–aldosterone system (RAAS) inhibitor drugs are associated with overexpression of ACE2. Entry into and replication of SARS‐CoV‐2 within human cells will be facilitated under these conditions and the resulting ACE2 downregulation will alter the previous equilibrium of the RAAS system and the clinical evolution of the disease. This effect may be more apparent in non‐hypertensive patients on RAAS inhibitor therapy. ACE2, angiotensin–converting enzyme 2; ACE, angiotensin–converting enzyme; Ang, angiotensin; MasR, mitochondrial assembly receptor; AT2R, angiotensin type 2 receptor; AT1R, angiotensin type 1 receptor.

The use of RAAS inhibitor drugs has been associated in animal models with an increase in ACE2 messenger RNA (mRNA) or protein levels and ACE2 activity, particularly for angiotensin receptor blockers (Ferrario et al., 2005; Ishiyama et al., 2004; Sukumaran et al., 2017). Overexpression of ACE2 alters the natural balance of the RAAS system by stimulating anti‐inflammatory and vasodilatory pathways. It has been shown, for example, that the anti‐hypertensive effects of conventional RAAS blockade are determined in part by the ability of these drugs to promote the circulating levels of Ang 1–7 (Stanziola et al., 1999). In the context of ACE2 overexpression, viral entry into and replication within human cells may be enhanced, and the previous equilibrium of the RAAS system may be altered favoring a more severe course of COVID‐19 in the group of patients on prior RAAS inhibitor treatment as suggested by the results of this study (Figure 2).

Of particular interest are the results obtained in the subgroup of non‐hypertensive patients on RAAS inhibitor therapy who appear to be more critical upon ICU admission and exhibit higher mortality. This result is all the more surprising as hypertension has been identified as a risk factor for the development of severe COVID‐19 (WHO, 2021). Potential unequal expression of ACE2 between hypertensive and non‐hypertensive patients may play a key role in explaining the differences in severity observed in this study. In this regard, basic research carried out in rat models with diet‐induced and spontaneous hypertension has linked increased blood pressure to reduced ACE2 mRNA expression and protein levels (Crackower et al., 2002; Yagil et al., 1996). Other studies have shown that persistent overexpression of ACE2 in neurological centers involved in blood pressure regulation results in significant attenuation of hypertension in spontaneously hypertensive animal models (Yamazato et al., 2007). In humans, the involvement of RAAS in the pathophysiology of cardiovascular disease has been extensively explored over the past decades and is currently well established (Ferrario, 2006; Nicholls et al., 2001). Some authors argue that decreased expression or activity of ACE2 may contribute to the development of hypertension by altering the counterbalance of the RAAS system (Ferrario, 2011; Wysocki et al., 2008). Unfortunately, the question of whether ACE2 is expressed differently in non‐hypertensive and hypertensive individuals remains unresolved. However, this hypothesis could explain the more severe forms of the disease observed in this study, adding to the effects of RAAS inhibitor drugs the fact that ACE2 might already be overexpressed in normotensive patients compared with hypertensive patients.

These results are in contrast to those of other studies. The largest, prospective cohort study published thus far, by Hippisley‐Cox et al. (2020), showed no significant increase in the risk of ICU admission and even a reduction in overall COVID‐19 in patients taking RAAS inhibitor drugs. On the other hand, RAAS blockers do not seem to be associated with increased mortality in COVID‐19 patients in the large meta‐analysis published by Kaur et al. (2021), although the study suggested a higher severity and need for ICU admission in patients using RAAS inhibitors. Interestingly, this study shows a reduction in mortality in hypertensive patients treated with RAAS inhibitor drugs. The study by Lopes et al. (2021) did not show a benefit in discontinuing RAAS inhibitor therapy in patients hospitalized with mild‐to‐moderate COVID‐19, but all patients in the study were hypertensive, and the risk appears to be more strongly correlated with long‐term use of these drugs. However, the role of RAAS inhibitor drugs in the more severe forms of COVID‐19 is not fully elucidated from our point of view, especially for non‐hypertensive patients as the present study highlights.

Finally, despite significant limitations due to small sample size, these findings warrant further targeted research and may be important for understanding the pathogenesis of SARS‐CoV‐2 and for identifying factors associated with the more severe course of disease it produces.

## AUTHOR CONTRIBUTIONS

All authors conceptualized the study and did the acquisition, analysis, or interpretation of the data. JJGM drafted the majority of the manuscript. All authors contributed to the drafting of the manuscript and critically revised it. All authors read and approved the final manuscript.

## FUNDING INFORMATION

No funding was obtained for this study.

## CONFLICT OF INTEREST

The authors declare that they have no competing interests. All data are available upon reasonable request.

## ETHICS STATEMENT

Local ethics committees approved the study. Regional Research Ethics Committee (CCER), Genève (Switzerland) – BASEC number: 2020–00917. Regional Research Ethics Committee (CCER), Vaud (Switzerland) – BASEC number: 2021–01299.
